# Non-Enzymatic Cell Expansion and Harvesting Using a Smart Thermo-Responsive Gel

**DOI:** 10.3390/gels11120962

**Published:** 2025-11-28

**Authors:** Zhiyu Yan, Nuno Honrado, Naiwen Tan, Md Anirban Jyoti, Linh Nguyen

**Affiliations:** 1 Division of Biomaterials and Tissue Engineering, Eastman Dental Institute, University College London, London NW3 2PF, UK; 2 UCL Eastman-Korea Dental Medicine Innovation Centre, Dankook University, Cheonan 31116, Republic of Korea

**Keywords:** alginate hydrogel beads, PNIPAAm, thermo-responsive polymer, non-enzymatic cell harvesting, cell therapy

## Abstract

Advanced cell-based therapies, including immunotherapy, regenerative medicine, and other biotechnological applications, require large quantities of viable mammalian cells for research and clinical use. Conventional enzymatic harvesting methods, such as trypsini-zation, can compromise cell integrity and reduce viability. This study investigates an al-ternative temperature-responsive approach using alginate beads incorporated with poly(N-isopropylacrylamide) (PNIPAAm), a polymer exhibiting a lower critical solution temperature (LCST) of approximately 32 °C. This system enables temperature-controlled cell detachment while preserving cellular structure and extracellular matrix components, thereby potentially improving post-harvest viability compared to trypsin treatment. Ho-mogeneous alginate hydrogel beads were synthesized using a standard infusion pump and ionically crosslinked with calcium cations. The beads were characterized by scanning electron microscopy (SEM) for morphology and by Fourier-transform infrared spectroscopy (FTIR), differential scanning calorimetry (DSC), and micro-computed tomography (µ-CT) for compositional and thermal analysis. Mouse fibroblast cells (L929 cell line) were cultured on the beads, and their proliferation and viability were assessed using CCK-8 and Live/Dead assays, demonstrating significant cell growth over seven days. The results suggest that PNIPAAm-modified alginate beads provide a promising, enzyme-free platform for efficient mammalian cell harvesting and delivery, with potential applications across advanced cell manufacturing and therapeutic technologies.

## 1. Introduction

Advances in cell-based therapies, including regenerative medicine and immunotherapy, have created a growing demand for efficient large-scale production of viable mammalian cells [1,2,3,4,5]. These therapeutic approaches rely on the expansion and harvesting of living cells that can repair damaged tissues, modulate immune responses, or deliver targeted treatments [6,7]. However, conventional enzymatic harvesting methods, particularly trypsinization can damage cell membranes and degrade the extracellular matrix (ECM), reducing cell viability and altering protein expression [8,9]. Therefore, developing gentle, scalable, and enzyme-free strategies for cell detachment is essential for improving the efficiency and reproducibility of biomanufacturing processes used in cell and gene therapy production.

Traditional cell culture techniques, such as monolayer and suspension systems, are limited by low scalability, high contamination risk, and cell damage during passaging [10,11,12]. Monolayer cultures require frequent enzymatic dissociation, while suspension cultures demand precise control of environmental parameters to prevent aggregation and necrosis [12,13,14,15]. Given that approximately 70% of biopharmaceuticals are produced using mammalian cell systems [16], optimizing culture and harvesting methods remains a major bottleneck for translational research and industrial-scale production.

One promising direction involves stimuli-responsive polymers, which undergo physical or chemical transitions in response to environmental cues such as temperature or pH [17,18]. Among these, poly(N-isopropylacrylamide) (PNIPAAm) is one of the most widely studied thermo-responsive polymers. It exhibits a lower critical solution temperature (LCST) of approximately 32 °C close to physiological conditions where it transitions from a hydrophilic, swollen state to a hydrophobic, collapsed one. This reversible phase behavior enables non-enzymatic, temperature-controlled cell detachment, as cells can adhere and proliferate at 37 °C and then spontaneously detach when cooled below the LCST [19,20,21,22,23,24]. Such functionality makes PNIPAAm an attractive candidate for enzyme-free harvesting in tissue engineering and biomanufacturing applications [19,22,24,25,26,27].

Despite these advantages, PNIPAAm alone lacks sufficient mechanical stability and bioactivity, which can limit its use in long-term cultures or clinical translation [28,29]. To overcome these shortcomings, researchers have explored hybrid materials combining PNIPAAm with natural polymers such as alginate, a biocompatible, biodegradable polysaccharide that forms hydrogels through ionic crosslinking with calcium ions [30,31,32]. Alginate hydrogels offer excellent water retention, permeability, and cell compatibility, making them a versatile platform for cell encapsulation and delivery [33]. When integrated with PNIPAAm, alginate-PNIPAAm composites exhibit both structural support and thermo-responsive functionality, enabling reversible control of cell adhesion and detachment under mild, physiologically relevant conditions [34,35,36,37].

The development of thermo-responsive alginate-PNIPAAm systems therefore represents a promising step toward scalable, non-invasive cell expansion and harvesting. By combining the mechanical and biological advantages of alginate with the phase-transition properties of PNIPAAm, these composite materials may preserve ECM integrity, enhance post-harvest viability, and minimize damage associated with enzymatic processing. This study aims to develop a novel thermo-responsive carrier platform based on alginate-PNIPAAm hydrogel beads for efficient and non-destructive mammalian cell expansion and harvesting. Through detailed material characterization and cell culture experiments, we seek to demonstrate that this approach offers a viable enzyme-free alternative to trypsinization, contributing to the advancement of next-generation cell manufacturing and therapeutic technologies.

## 2. Results and Discussion

**Figure 1:**
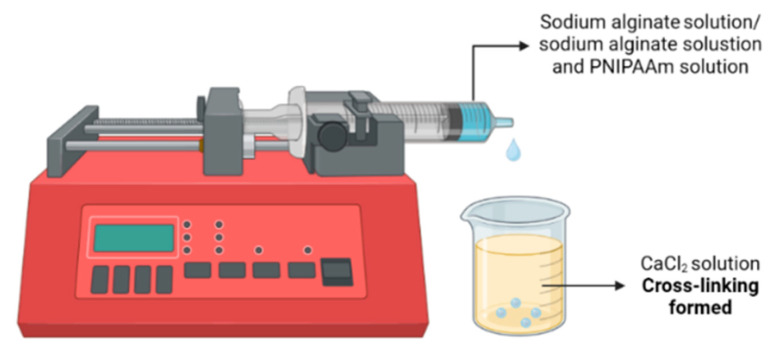
Schematic illustration of the preparation of Alginate Beads with and without PNIPAAm.

This study aimed to develop and evaluate thermo-responsive alginate-PNIPAAm hybrid beads as potential carriers for cell expansion and enzyme-free harvesting. The fabrication process was optimized to achieve a balance between porosity, mechanical integrity, and temperature sensitivity. As illustrated schematically in Figure 1, the alginate-PNIPAAm solutions were dispensed using a syringe pump into a calcium chloride (CaCl_2_) crosslinking bath to ensure uniform bead formation. Preliminary screening of PNIPAAm concentrations demonstrated that lower polymer loadings (<0.5 g) produced weak thermo-responsive behavior, whereas higher concentrations (>1.0 g) resulted in bead fragility and deformation during crosslinking. Therefore, 0.5 g and 1.0 g PNIPAAm were selected as representative low and high loadings for detailed evaluation.

### 2.1. Thermoresponsive Behavior and Cloud Point of PNIPAAm

**Figure 2:**
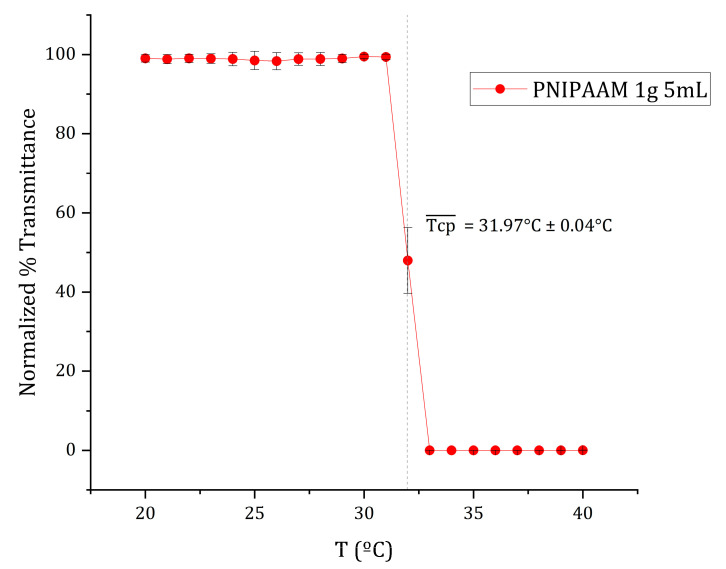
Mean normalized transmittance vs. temperature curve of 1 g/5 mL PNIPAAM aqueous solution at a thermal ramp rate of ca. 0.5 °C/min. The cloud point (Tcp) of each replicate was defined as 50% of the maximum transmittance and estimated through sigmoidal curve fitting using OriginPro 2023b software. The average cloud point, $\bar{Tcp}$, as indicated by the dotted line, was calculated from the average of the individual curves’ cloud points. Data presented as mean ± SD (*n* = 3).

The thermoresponsive transition of amine-terminated poly(N-isopropylacrylamide) (PNIPAAm-NH_2_) was characterized by monitoring temperature-dependent changes in transmittance during a controlled heating ramp (Figure 2). The 1 g/5 mL aqueous PNIPAAm solution maintained a stable transmittance of approximately 73% from 20 °C to 31 °C, indicating a well-hydrated, coil-like polymer conformation. Between 31 °C and 33 °C, transmittance sharply decreased to nearly 0%, marking a coil-to-globule phase transition. The cloud-point temperature (Tcp) was defined as the temperature corresponding to 50% of the maximum transmittance [38] and was determined by sigmoidal curve fitting to be 32 °C.

Above 33 °C, the solution became completely opaque due to polymer aggregation, consistent with the lower critical solution temperature (LCST) behavior of PNIPAAm in water. The obtained Tcp (~32 °C) agrees with previously reported LCST values for PNIPAAm [19,22,24]. This transition reflects the polymer’s reversible coil-to-globule conformational change, driven by disruption of hydrogen bonding and enhanced hydrophobic interactions. The LCST observed aligns closely with physiological temperature, validating PNIPAAm’s suitability for temperature-controlled cell culture and harvesting systems [20,21]. The sharpness of this transition also suggests good molecular uniformity and minimal copolymer impurities in the material.

### 2.2. Characterization of Alginate and Alginate/PNIPAAm Beads

#### 2.2.1. Scanning Electron Microscopy (SEM)

**Figure 3:**
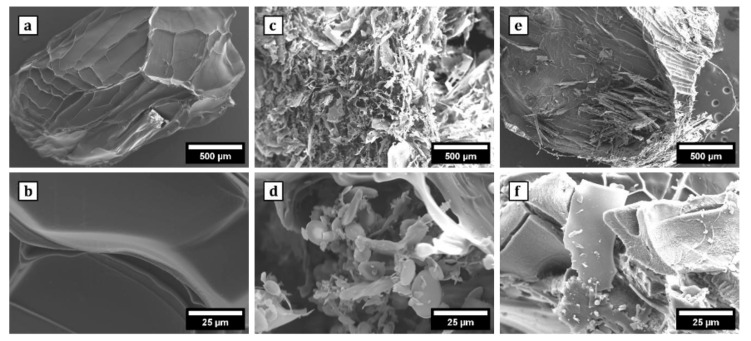
SEM images of the surface morphology of Alg beads at 50× (**a**) and 1000× (**b**); Alg/0.5 g PNIPAAm beads at 50× (**c**) and 1000× (**d**); Alg/1 g PNIPAAm beads at 50× (**e**) and 1000× (**f**). It was observed that the surface of Alg beads was relatively smooth and the surface of Alg/0.5 g PNIPAAm beads and Alg/1 g PNIPAAm beads showed a porous structure.

SEM imaging revealed distinct surface morphologies among Alg, Alg/0.5g PNIPAAm, and Alg/1g PNIPAAm beads (Figure 3). Pure alginate beads displayed relatively smooth, compact surfaces, whereas PNIPAAm incorporation produced visibly rougher, porous topographies. The pores were more pronounced in Alg/1g PNIPAAm beads, suggesting increased polymer phase separation and structural heterogeneity. The enhanced roughness and porosity are expected to provide greater surface area for cell attachment and nutrient exchange, an attribute advantageous for 3D cell culture. The pore diameters (50–100 µm) are favorable for mammalian cell attachment and nutrient transport, as supported by previous work showing that freeze-dried alginate scaffolds create interconnected networks conducive to cell adhesion [39].

#### 2.2.2. Fourier Transform Infrared Spectroscopy (FTIR)

**Figure 4:**
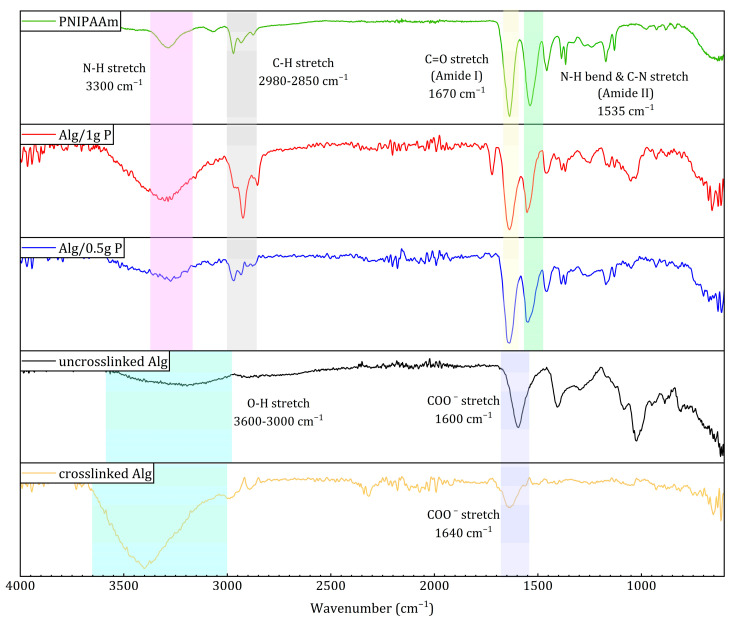
ATR-FTIR spectra of PNIPAAm, Alg/1 g PNIPAAm, Alg/0.5 g PNIPAAm beads, uncrosslinked alginate, and crosslinked alginate beads.

FTIR spectra (Figure 4) confirmed successful integration of PNIPAAm within the alginate matrix. Characteristic PNIPAAm absorption bands appeared at ~1670 cm^−1^ (amide I, C=O stretching) and ~1535 cm^−1^ (amide II, N–H bending + C–N stretching), which were absent in pure alginate. Additionally, the O–H stretching band (3600–3000 cm^−1^) in alginate became sharper and shifted toward ~3300 cm^−1^ in the composite beads, consistent with N–H stretching of secondary amides. Minor peaks at 2980–2850 cm^−1^ correspond to symmetric and asymmetric C–H stretching from PNIPAAm’s isopropyl groups. These spectral changes collectively indicate the presence of PNIPAAm in the alginate network without chemical degradation. Shifts in carboxyl and hydroxyl stretching bands indicated hydrogen bonding and ionic interactions between alginate and PNIPAAm chains, a feature common in polysaccharide–amide composites [40,41].

#### 2.2.3. Differential Scanning Calorimetry (DSC)

**Figure 5:**
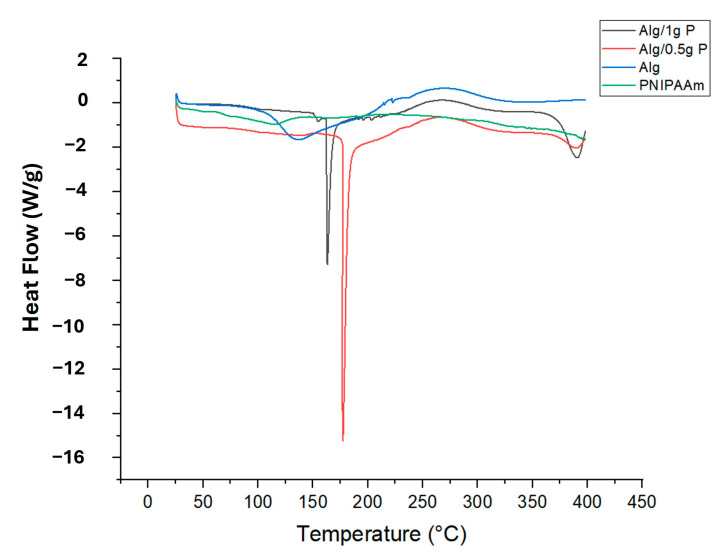
DSC diagram of PNIPAAm, Alg beads, Alg/0.5 g PNIPAAm beads and Alg/1 g PNIPAAm beads.

DSC thermograms (Figure 5) showed clear differences in thermal behavior between the materials. Alginate beads exhibited a broad endothermic transition near 130 °C, attributable to moisture loss and polymer softening. PNIPAAm incorporation shifted this transition to ~160 °C (Alg/0.5 g PNIPAAm) and ~175 °C (Alg/1 g PNIPAAm), demonstrating enhanced thermal stability due to intermolecular interactions between PNIPAAm and alginate chains. The sharper peaks at higher temperatures further support successful polymer blending and structural reinforcement.

The persistent exothermic peak at ≈250 °C indicates that polymer degradation characteristics remain unaffected. It is also critical to reflect on how the intrinsic functional groups of alginate may influence the thermo-responsive behavior of PNIPAAm when embedded in a hybrid matrix. The carboxylate (–COO^−^) and hydroxyl (–OH) groups present in alginate can engage in hydrogen bonding or ionic interactions with the amide groups of PNIPAAm, thereby altering the hydration shell, chain mobility, and ultimately the lower critical solution temperature (LCST) and the amplitude of the thermo-switching response. Recent reviews highlight that such polysaccharide–thermoresponsive polymer interfaces can cause measurable shifts in transition temperature and response sharpness, depending on grafting density, ionic strength, and cross-linking context [42]. In our alginate-PNIPAAm beads, the observed LCST-proximate behavior (~32 °C) therefore suggests that although some interaction may occur, it does not preclude effective thermo-responsive detachment under physiological conditions. Nonetheless, a more systematic investigation such as variable ionic strength testing, DSC in buffered media, or rheological evaluation across PNIPAAm loading is warranted to quantify the influence of alginate-PNIPAAm coupling on transition behavior.

#### 2.2.4. Microcomputed Tomography (Micro-CT)

**Figure 6:**
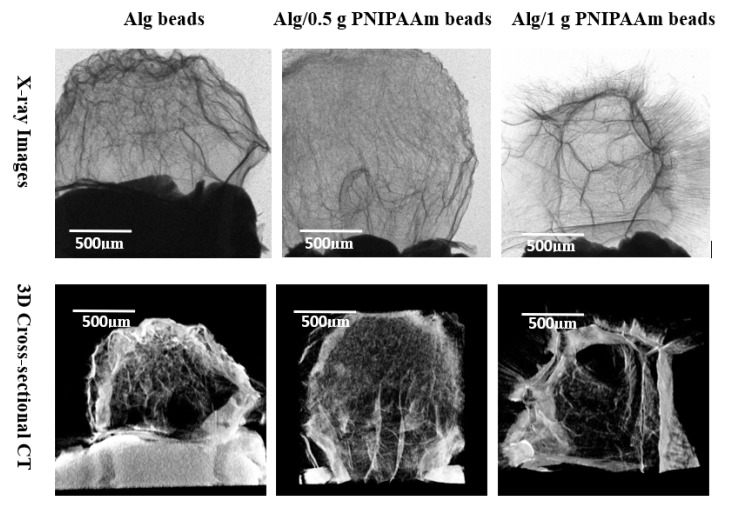
Both X-ray (2D) and cross-sectional CT (3D) images of Alg beads, Alg/0.5 g PNIPAAm beads and Alg/1 g PNIPAAm beads showing internal patches and fibrotic orientation leading to roughness. The surfaces of Alg/0.5 g PNIPAAm beads and Alg/1 g PNIPAAm beads exhibit clear fibrous structures. Alg beads have a typical surface of freeze-dried hydrogel pattern.

Micro-CT reconstructions (Figure 6) revealed well-defined, interconnected porous networks within all bead types. Pure alginate beads displayed uniform internal density typical of freeze-dried hydrogels, while PNIPAAm-modified beads exhibited fibrous, irregular inner architectures with enhanced textural contrast. The increased internal connectivity, particularly in Alg/1 g PNIPAAm beads, suggests improved diffusion pathways that may facilitate efficient nutrient transport during cell culture.

All samples retained an overall spherical shape, confirming the reproducibility of the fabrication process. The X-ray images (upper panel) revealed distinct internal textural differences among the bead types: the pristine alginate beads displayed a relatively homogeneous internal network typical of freeze-dried hydrogels, whereas the Alg/0.5 g PNIPAAm and Alg/1 g PNIPAAm beads exhibited more prominent fibrous and patch-like features extending throughout the structure. The density gradients and fibrotic streaks observed in the PNIPAAm-modified beads suggest localized polymer-rich domains formed during gelation. Micro-CT cross-sectional imaging (lower panel) corroborated these observations, showing that the incorporation of PNIPAAm produced an increasingly porous and interconnected internal network compared with the denser morphology of pure alginate beads. The Alg/1 g PNIPAAm beads demonstrated the most pronounced internal channels and fibrotic orientations, indicative of enhanced phase separation and structural roughness. These internal microchannels likely contribute to improved permeability and potential diffusion of nutrients during cell culture. In contrast, the smoother, less porous alginate beads exhibited a more uniform but compact internal texture, consistent with their lower polymer heterogeneity. Collectively, the imaging results confirm that PNIPAAm integration increases microstructural complexity and interconnectivity without compromising overall shape uniformity or structural cohesion.

### 2.3. Cell Viability and Proliferation Assessment

**Figure 7:**
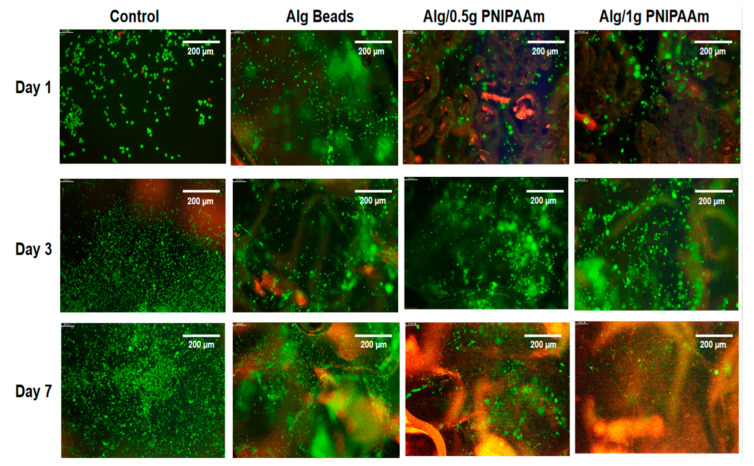
Cell proliferation study using LIVE/DEAD assay. Representative fluorescence images (Leica Instruments, Milton Keynes, UK) of L929 cells cultured for 1, 3, and 7 days on different sur-faces: control (culture medium), alginate (Alg) beads, Alg/0.5 g PNIPAAm beads, and Alg/1 g PNIPAAm beads. Live cells are stained green and dead cells red. Scale bar (at the top right of each image) = 200 µm.

**Figure 8:**
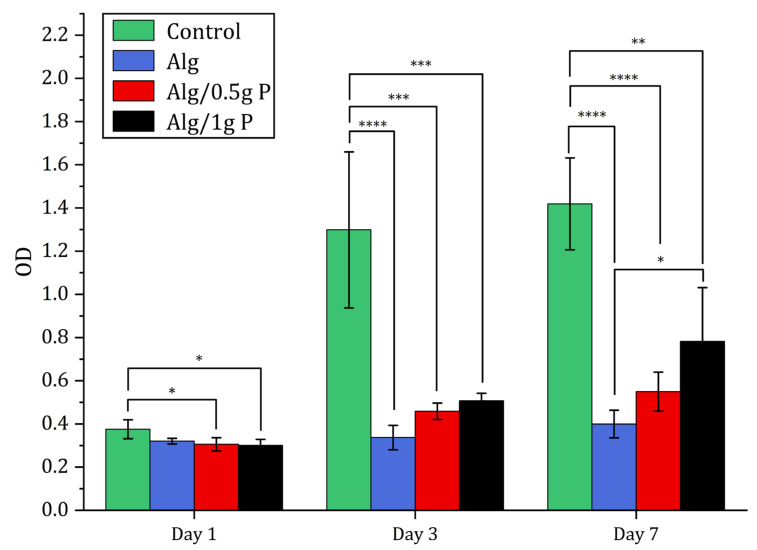
Cell proliferation study using CCK-8. CCK-8 results of L929 cells seeded on TCP (tissue culture plate) (control), Alg beads, Alg/0.5 g PNIPAAm beads and Alg/1 g PNIPAAm beads after 1, 3 and 7 days. Data presented as mean ± SD (*n* = 4). Statistical analysis across different groups was performed with one-way ANOVA followed by Tukey’s post hoc test, * *p* < 0.05, ** *p* < 0.01, *** *p* < 0.001, **** *p* < 0001.

The biocompatibility of the beads was evaluated using L929 fibroblasts through LIVE/DEAD and CCK-8 assays (Figure 7 and Figure 8). Fluorescence microscopy demonstrated predominantly green (live) staining with minimal red (dead) signal for all formulations over 7 days, indicating negligible cytotoxicity. Cells progressively distributed across the bead surfaces, reflecting favorable adhesion within the porous structures.

Quantitative metabolic analysis via CCK-8 showed a time-dependent increase in absorbance from day 1 to day 7 for all groups. Notably, cell proliferation on Alg/0.5 g PNIPAAm and Alg/1 g PNIPAAm beads was significantly higher (*p* < 0.05) than on Alg beads alone at day 7, confirming that PNIPAAm incorporation promotes enhanced cell growth. The Alg/1 g PNIPAAm formulation yielded the highest metabolic activity, correlating with its greater surface roughness and pore connectivity.

Although overall viability was slightly lower than that of the 2D control, the majority of cells remained viable, confirming that the Ca^2+^-crosslinked alginate–PNIPAAm network was non-toxic. It is noteworthy that the control group represents cells cultured on a 2D tissue culture plate, whereas the alginate-based systems provided a 3D microenvironment. Therefore, while the control serves as a reference for baseline viability, direct quantitative comparison should be interpreted with caution, as 2D and 3D growth environments inherently differ in diffusion, oxygenation, and surface contact. Across all bead formulations, viable cells (green fluorescence) were evident at each time point, with only minimal red fluorescence indicating dead cells. Cell density increased from Day 1 to Day 7, particularly on Alg/1 g PNIPAAm beads, suggesting that the incorporation of a higher PNIPAAm content enhanced surface properties conducive to cell attachment and metabolic activity. The likely mechanism involves the combined effects of surface roughness and an optimized hydrophilic–hydrophobic balance near physiological temperature, which favor transient cell–substrate interactions and promote proliferation. Quantitative CCK-8 assays corroborated the qualitative fluorescence observations: metabolic activity increased progressively over the 7-day period for all bead groups, with the Alg/1 g PNIPAAm beads exhibiting the highest proliferation among the 3D constructs (Figure 8). These findings are consistent with earlier reports that PNIPAAm-modified substrates enhance cell adhesion and spread under physiological conditions due to temperature-responsive surface rearrangements [20,21,43].

### 2.4. Cell Detachment Experiment

**Figure 9:**
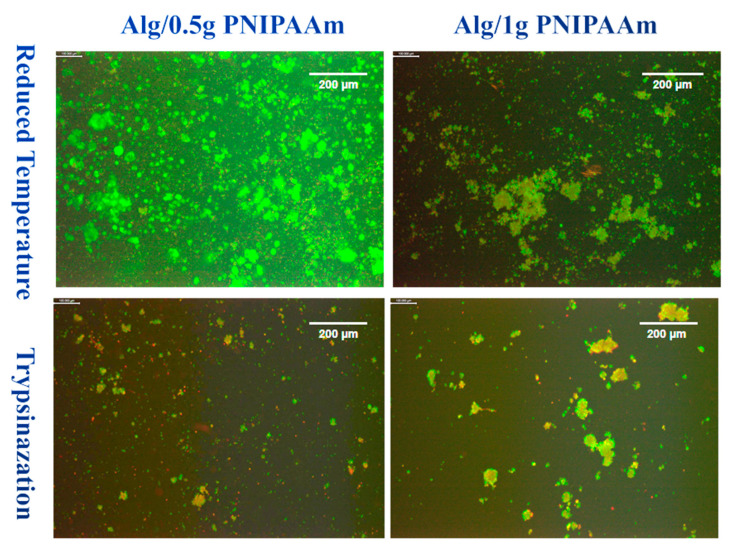
LIVE/DEAD images of L929 cells after 14 days of incubation on Alg/0.5 g PNIPAAm beads and Alg/1 g PNIPAAm beads surface and harvested by two different methods. Green = live cells; Red = dead cells. Differences in green/red shades reflect natural variations in cell viability and staining intensity. Scale bar (at the top right of each image) = 200 µm.

To visualize cell detachment behavior under different harvesting conditions, L929 fibroblasts were cultured for 14 days on Alg/0.5 g PNIPAAm and Alg/1 g PNIPAAm beads. Following incubation, cells were detached using either a temperature-reduction method or enzymatic treatment with trypsin, and evaluated by fluorescence microscopy (Figure 9).

Under reduced temperature conditions, both bead types showed extensive areas of green fluorescence, indicating a predominance of viable cells after detachment. The Alg/1 g PNIPAAm beads displayed larger and denser aggregates of green-stained cells, whereas Alg/0.5 g PNIPAAm samples exhibited more finely dispersed clusters. In contrast, trypsin-treated samples showed markedly fewer fluorescent regions and a greater presence of red staining, indicating an increased number of non-viable cells.

**Figure 10:**
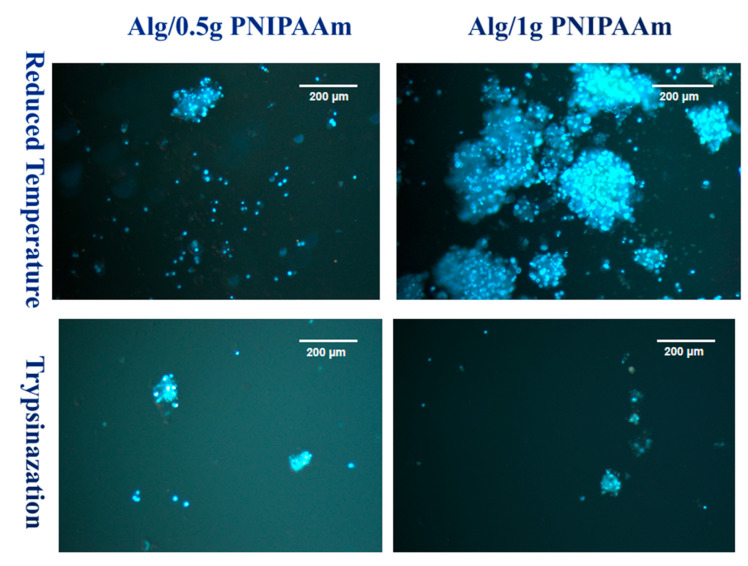
DAPI staining images of L929 cells after 14 days of incubation on Alg/0.5 g PNIPAAm beads and Alg/1 g PNIPAAm beads surface and harvested by two different methods.

Complementary DAPI staining (Figure 10) confirmed the differences in detached cell distribution. After temperature reduction, numerous, blue-stained nuclei were visible across both formulations, with particularly large aggregates observed on Alg/1 g PNIPAAm beads. Following trypsinization, fewer nuclei were detected, and the cell clusters appeared smaller and more sparsely distributed. These observations demonstrate clear qualitative variations in cell density and morphology among the different detachment conditions and PNIPAAm loadings.

After 14 days, significant cell proliferation was present in the group of cells. However, more cells were collected using the temperature-reducing method and relatively fewer dead cells were collected using the temperature-reducing method compared to those collected using trypsin.

Temperature-induced detachment assays demonstrated efficient cell recovery from PNIPAAm-containing beads when cooled below the LCST (Figure 9 and Figure 10). Cells detached as intact clusters with minimal red fluorescence, whereas trypsinized samples exhibited greater cell death. DAPI staining confirmed the viability and structural integrity of the detached cells. This temperature-mediated detachment aligns with previous PNIPAAm-based systems that enable enzyme-free release of cells while preserving extracellular matrix proteins [43,44]. Cell detachment was qualitatively observed for both Alg/0.5 g and Alg/1 g PNIPAAm beads upon temperature reduction. While Figure 9 suggests a higher detachment in the Alg/1 g PNIPAAm formulation, this variation likely reflects sample heterogeneity including local porosity and cell distribution rather than a consistent quantitative trend. Increasing PNIPAAm content can enhance adhesion during culture due to improved hydrophobic interactions but may transiently hinder detachment because of denser polymer collapse near the LCST. These findings confirm the feasibility of temperature-triggered cell release while underscoring the need for future systematic quantification of detachment efficiency and PNIPAAm content optimization. It is important to note that the observed differences in cell detachment efficiency between 0.5 g and 1 g PNIPAAm beads are preliminary and qualitative. These results reflect the proof-of-concept nature of this study rather than statistically validated performance. Future investigations will incorporate image-based cell counting and quantitative detachment assays to optimize PNIPAAm concentration and correlate detachment efficiency with polymer hydration dynamics and mechanical stiffness near the LCST.

It is also important to consider that mammalian cell culture is routinely performed in buffered media such as DMEM, which contains various ionic species (e.g., Na^+^, K^+^, Ca^2+^, Cl^−^, HCO_3_^−^) and serum proteins that can significantly influence the LCST of PNIPAAm. The ionic composition, osmolarity, and presence of salts can shift the transition temperature through ion-specific (Hofmeister) effects, altering polymer-water interactions and chain hydration dynamics. Recent studies have demonstrated that phosphate and bicarbonate ions can lower the LCST by screening hydrogen bonding and promoting hydrophobic collapse of PNIPAAm chains [45,46]. Consequently, the thermoresponsive behavior observed in deionized-water systems may differ quantitatively under physiological conditions. The successful temperature-triggered cell detachment observed here suggests that, despite such effects, the LCST of the alginate-PNIPAAm composite remains within the biologically relevant range (approximately 30–35 °C), ensuring effective thermoresponsive performance in standard culture media [45,46].

## 3. Conclusions

Thermo-responsive alginate-PNIPAAm hybrid beads were successfully fabricated and characterized as enzyme-free cell carriers. The incorporation of PNIPAAm introduced porosity and enhanced thermal stability while maintaining spherical integrity and LCST behavior near 32 °C (Figure 2 and Figure 5). FTIR confirmed characteristic amide linkages (Figure 4), and SEM and Micro-CT imaging (Figure 3 and Figure 6) verified uniform, interconnected porous networks conducive to cell growth. Biological assays demonstrated that fibroblasts cultured on these hybrid beads remained highly viable and proliferative (Figure 7 and Figure 8). Upon cooling below the LCST, cells detached spontaneously and retained intact nuclei (Figure 9 and Figure 10), showing that temperature-triggered recovery offers a gentler alternative to enzymatic trypsinization. Beads containing 1 g PNIPAAm exhibited superior thermo-responsiveness and detachment efficiency compared with 0.5 g formulations, emphasizing the compositional tunability of this system. These results collectively demonstrate that alginate-PNIPAAm hybrid beads exhibit favorable structural, thermal, and biological performance characteristics suitable for thermo-responsive cell culture. The LCST close to physiological temperature ensures compatibility with mammalian systems, while the porous morphology promotes cell adhesion and nutrient diffusion. Future work should include quantitative evaluation of detachment kinetics, rheological assessment of temperature-dependent viscoelastic properties, and exploration of LCST shifts in complex media containing salts and proteins, which may influence polymer hydration and cell–substrate interactions. Such investigations will strengthen the translational potential of this system for scalable and gentle cell harvesting applications in regenerative medicine.

## 4. Materials and Methods

### 4.1. Materials

Alginic acid sodium salt from brown algae (sodium alginate, CAS 9005-38-3), sodium chloride (NaCl, CAS 7647-14-5), calcium chloride (CaCl_2_, CAS 10043-52-4), and amine-terminated poly(N-isopropylacrylamide) (PNIPAAm-NH_2_, Product No. 724823) were purchased from Sigma-Aldrich (Gillingham, UK). All reagents were used as received without further purification. Deionized water was used in all experiments.

### 4.2. Determination of PNIPAAm Cloud Point via Dynamic Light Scattering (DLS)

The thermoresponsive behavior of PNIPAAm-NH_2_ was evaluated by determining its cloud-point temperature (Tcp) from temperature-dependent transmittance measurements. PNIPAAm-NH_2_ (1.0 g) was dissolved in 5.0 mL deionized water with continuous stirring at room temperature until a homogeneous solution was obtained, then filtered through a 0.45 µm syringe filter. Measurements were performed in triplicate using a Litesizer 500 spectrophotometer (Anton Paar GmbH, Graz, Austria) equipped with a temperature-controlled cuvette holder. Each sample was heated from 20 °C to 40 °C in 1 °C increments at approximately 0.5 °C min^−1^, equilibrating 1 min at each step. Transmittance was recorded at a fixed scattering angle of 90°. The cloud point was defined as the temperature corresponding to a 50% decrease in transmittance, indicating the coil-to-globule phase transition of PNIPAAm chains. The inflection point was determined by sigmoidal curve fitting using OriginPro 2023b (OriginLab, Northampton, MA, USA).

### 4.3. Preparation of Alginate and Alginate/PNIPAAm Beads

#### 4.3.1. Preparation of Solutions

A 1% (*w*/*v*) sodium alginate solution was prepared in 0.9% (*w*/*v*) NaCl by dissolving 0.2 g sodium alginate and 0.18 g NaCl in 20 mL distilled water. The solution was heated to ≈ 80 °C and stirred magnetically until completely dissolved. For PNIPAAm-containing formulations, PNIPAAm-NH_2_ (0.5 g or 1.0 g) was first dissolved in 5 mL cold distilled water (approximately 4 °C) and then mixed with 15 mL of the 1% alginate solution to obtain Alg/0.5 g PNIPAAm and Alg/1 g PNIPAAm mixtures, respectively. A 0.1 M calcium chloride (CaCl_2_) crosslinking solution was prepared by dissolving 11.1 g CaCl_2_ in 1 L distilled water.

#### 4.3.2. Bead Preparation and Cross-Linking

The alginate and alginate/PNIPAAm solutions were loaded into 5 mL syringes and dispensed dropwise into 0.1 M CaCl_2_ using a syringe pump (Standard Infuse Pump 11 Elite, Harvard Apparatus, Cambridge, MA, USA) at 1 mL min^−1^. Crosslinking was allowed to proceed for 15–20 min. Beads were collected using pre-moistened filter paper, washed five times with distilled water, frozen overnight at −80 °C (New Brunswick ultra-low temperature freezer), and lyophilized for 24 h using a freeze-dryer (Alpha 1-2 LDplus, Martin Christ, Osterode, Germany).

### 4.4. Characterisation of Material

#### 4.4.1. Scanning Electron Microscopy (SEM)

The morphological structures of the freeze-dried alginate beads (Alg beads) and alginate/poly(N-isopropylacrylamide) beads (Alg/PNIPAAm beads) were examined using a scanning electron microscope (SEM) (Zeiss EVO HD, Jena, Germany). Both bead types were bisected to expose the cross-linked sections for SEM analysis. Images were captured at a scan speed of 8. Samples were coated with a 5% palladium and 95% gold mixture using a SEM Coating Unit E5000 (Polaron Equipment Limited, Watford, UK).

#### 4.4.2. Fourier Transform Infrared Spectroscopy (FTIR)

ATR-FTIR spectra were obtained for PNIPAAm powder, sodium alginate, Alg beads, and Alg/PNIPAAm beads using a Spectrum One FTIR spectrometer (PerkinElmer, Llantrisant, UK) over 4000–500 cm^−1^. Spectra were analyzed to confirm characteristic functional groups and polymer interactions.

#### 4.4.3. Differential Scanning Calorimetry (DSC)

Samples (3–5 mg each) of Alg, Alg/PNIPAAm (0.5 g and 1 g), and PNIPAAm powder were sealed in aluminum pans and analyzed using a DSC 25 calorimeter (TA Instruments, New Castle, DE, USA). Heating was performed from 25 °C to 400 °C at 10 °C min^−1^ under a nitrogen atmosphere. Thermal transitions and stability were evaluated from endothermic and exothermic peaks.

#### 4.4.4. Micro-Computed Tomography (Micro-CT)

Internal structures of Alg and Alg/PNIPAAm beads were examined by micro-computed tomography (SkyScan 1275, Bruker microCT, Kontich, Belgium). Three-dimensional reconstructions and quantitative analyses were performed using NRecon (v1.8.x), CTAn (v1.20.x), and CTvox (v3.3.x) software (Bruker, Belgium).

### 4.5. In Vitro Experiments

#### 4.5.1. Cell Culture

Mouse fibroblast cells (L929, ATCC CCL-1) were cultured in Dulbecco’s Modified Eagle Medium (DMEM, Sigma-Aldrich, Gillingham, UK) supplemented with 10% fetal bovine serum (FBS), 100 U mL^−1^ penicillin, and 100 µg mL^−1^ streptomycin. Cultures were maintained at 37 °C in a humidified 5% CO_2_ incubator.

#### 4.5.2. Cell Viability and Proliferation

Cell viability and proliferation on Alg, Alg/0.5 g PNIPAAm, and Alg/1 g PNIPAAm beads were assessed using the LIVE/DEAD^®^ Viability/Cytotoxicity kit (Thermo Fisher Scientific, Leicester, UK) and Cell Counting Kit-8 (CCK-8, Dojindo, Japan).

For LIVE/DEAD analysis, L929 cells were seeded onto sterilized beads in 48-well plates (6 mm wells, *n* = 2) at 5 × 10^4^ cells mL^−1^ and incubated for 1, 3, and 7 days. Samples were washed with phosphate-buffered saline (PBS), stained with 2 µM calcein-AM and 4 µM EthD-1 for 20 min at room temperature, and imaged by fluorescence microscopy (Leica Instruments, Milton Keynes, UK).

For CCK-8 assays, cells were seeded under identical conditions (*n* = 4). At each time point, samples were washed with PBS and incubated with 500 µL fresh medium containing 20 µL CCK-8 reagent for 3 h at 37 °C. Absorbance was measured at 460 nm using a microplate reader (Infinite M200, Tecan Ltd., Reading, UK).

#### 4.5.3. Cell Detachment Experiment

Thermoresponsive detachment was evaluated using L929 cells seeded on Alg/0.5 g PNIPAAm and Alg/1 g PNIPAAm beads (5 × 10^4^ cells mL^−1^) in cell-repellent 24-well plates (13 mm wells, *n* = 2). After 14 days of culture, one set was exposed to cold medium (4 °C, 10 min) to induce detachment, while a control group underwent enzymatic detachment with 0.25% trypsin. Detached cells were collected, stained with LIVE/DEAD^®^ and DAPI (F6057-20ML, Sigma-Aldrich, Gillingham, UK), and visualized using a fluorescence microscope (Leica Instruments, Milton Keynes, UK).

### 4.6. Statistical Analysis

All experiments were performed in triplicate unless otherwise stated. Quantitative data are expressed as mean ± standard deviation (SD). Statistical significance was determined using one-way analysis of variance (ANOVA) followed by Tukey’s post hoc test (*p* < 0.05 considered significant). Graphs were generated using OriginPro 2023b (OriginLab, Northampton, MA, USA), and images processed using ImageJ (1.53c) (NIH, Bethesda, MD, USA).

## Data Availability

The original contributions presented in this study are included in the article. Further inquiries can be directed to the corresponding author.
